# Applying dynamic contrast-enhanced MRI tracer kinetic models to differentiate benign and malignant soft tissue tumors

**DOI:** 10.1186/s40644-024-00710-x

**Published:** 2024-05-21

**Authors:** Aixin Gao, Hexiang Wang, Xiuyun Zhang, Tongyu Wang, Liuyang Chen, Jingwei Hao, Ruizhi Zhou, Zhitao Yang, Bin Yue, Dapeng Hao

**Affiliations:** 1 Department of Radiology, The Affiliated Hospital of Qingdao University, 16 Jiangsu Road, Qingdao, Shandong China; 2 Department of Clinic Lab, Qingdao Cancer Hospital, Qingdao, Shandong China; 3 Fisca Healthcare (nanjing) Co., Ltd, Nanjing, Jiangsu China; 4 Department of Bone Oncology, The Affiliated Hospital of Qingdao University, 16 Jiangsu Road, Qingdao, Shandong China

**Keywords:** Soft tissue tumors, Dynamic contrast-enhanced MRI, Diagnosis

## Abstract

**Background:**

To explore the potential of different quantitative dynamic contrast-enhanced (qDCE)-MRI tracer kinetic (TK) models and qDCE parameters in discriminating benign from malignant soft tissue tumors (STTs).

**Methods:**

This research included 92 patients (41females, 51 males; age range 16–86 years, mean age 51.24 years) with STTs. The qDCE parameters (K^trans^, K_ep_, V_e_, V_p_, F, PS, MTT and E) for regions of interest of STTs were estimated by using the following TK models: Tofts (TOFTS), Extended Tofts (EXTOFTS), adiabatic tissue homogeneity (ATH), conventional compartmental (CC), and distributed parameter (DP). We established a comprehensive model combining the morphologic features, time-signal intensity curve shape, and optimal qDCE parameters. The capacities to identify benign and malignant STTs was evaluated using the area under the curve (AUC), degree of accuracy, and the analysis of the decision curve.

**Results:**

TOFTS-K^trans^, EXTOFTS-K^trans^, EXTOFTS-V_p_, CC-V_p_ and DP-V_p_ demonstrated good diagnostic performance among the qDCE parameters. Compared with the other TK models, the DP model has a higher AUC and a greater level of accuracy. The comprehensive model (AUC, 0.936, 0.884–0.988) demonstrated superiority in discriminating benign and malignant STTs, outperforming the qDCE models (AUC, 0.899–0.915) and the traditional imaging model (AUC, 0.802, 0.712–0.891) alone.

**Conclusions:**

Various TK models successfully distinguish benign from malignant STTs. The comprehensive model is a noninvasive approach incorporating morphological imaging aspects and qDCE parameters, and shows significant potential for further development.

**Supplementary Information:**

The online version contains supplementary material available at 10.1186/s40644-024-00710-x.

## Introduction

Soft tissue tumors (STTs) form an extraordinarily heterogeneous group [1]. The distinction between benign and malignant tumors is crucial because malignant lesions frequently necessitate wide-margin therapy and adjuvant cytotoxic therapy, whereas benign lesions require only serial imaging and monitoring [2–7]. However, the rarity of STTs as well as their overlapping histologic and radiologic characteristics impedes accurate classification [1].

Dynamic contrast-enhanced MRI (DCE-MRI) is a well-established technology for evaluating tumor blood microcirculation and vessel permeability [8]. In the majority of tissues, the MR tracer distributes over an intra- and extravascular extracellular space (EES), with a bi-directional exchange of tracer across the barrier between the spaces [9]. Several tracer kinetic (TK) models have been developed to obtain quantitative DCE (qDCE) parameters characterizing the tumor microcirculation state. Compartmental and spatially distributed models are the two primary types of models used in dynamic perfusion data analysis [10, 11]. Compartmental models include Tofts (TOFTS), extended Tofts (EXTOFTS), and conventional compartmental (CC) models. Spatially distributed models comprise the adiabatic approximated tissue homogeneity model (ATH) and the distributed parameter (DP) model [10]. TOFTS, the most widely used model for analyzing DCE - MRI data, assumes a negligible contribution of intravascular contrast agents to the total tissue concentration [11, 12]. EXTOFTS attempts to account for a vascular plasma space [11]. Classical TK models such as EXTOFTS have been used to distinguish between benign and malignant STTs [13–15]. However, these two models are limited by using only a single constant transfer parameter (K^trans^) to model transport, and thereby cannot distinguish between the transport of tracer molecules in blood vessels and the exchange process of tracer molecules between blood vessels and tissues [16].

Advanced pharmacokinetic models, such as CC, ATH, and DP, have been proposed, providing a more accurate explanation of tracer transport in the tissue microenvironment, and rendering derived parameters that better describe the tumor tissue microenvironment [9, 11, 16, 17]. These three models are concerned with two transport processes: intravascular perfusion of tracer molecules, and osmotic exchange inside and outside the vessel through the vessel wall. By using individual parameters for blood flow (F) and permeability-surface area product (PS), they successfully describe these processes [16]. There are differences in the three models: CC posits that tracer concentrations are evenly distributed between the inner and outer vascular spaces, whereas the ATH suggests that tracer concentrations fluctuate over time and space within the intravascular space [10, 11, 17]. DP contends that tracer concentrations vary temporally and spatially in both the intravascular and extravascular extracellular spaces [10, 11]. These advanced technologies were already applied to cervical cancer [18, 19], endometrial cancer [20], and glioma [16]. They achieved adequate performance in assessing the microcirculation pattern in cervix cancer tissue, evaluating preoperative risk for endometrial cancer, and assessing glioma IDH mutation status, respectively. However, advanced TK models have not been researched for STTs classification.

The effectiveness of different TK models in distinguishing benign from malignant STTs differs, and it is yet to be explored if a comprehensive model combining traditional imaging features and qDCE parameters can enhance the diagnosis performance. In this study, we aimed to compare the performance of different TK models in differentiating benign and malignant STTs. We also assessed if the comprehensive model enhanced diagnostic capability and enabled treatment plan optimization.

## Methods

### Patients

This research was authorized by our institution’s ethical committee. Written informed consent was obtained from each patient before the MRI examination. We selectively recruited ninety-two patients who were pathologically diagnosed with STTs between January 2017 and September 2022 (51 males and 41 females, 16 to 86 years old with mean age 51.24 years). Inclusion criteria include: (1) all patients had undergone a 3.0T DCE MRI scan; (2) no chemotherapy or radiotherapy before surgery; (3) patients with histopathologically proven STTs. Exclusion criteria include: (1) poorly vascularized tumors like lipoma and well-differentiated liposarcoma; (2) inadequate image quality due to motion artifacts; (3) intermediate tumors such as myoepithelioma. All lesions were divided into groups of benign or malignant tumors based on the pathological categorization of soft-tissue tumors by the World Health Organization (2020) [21].

### MRI acquisition

The MRI examinations were obtained using a 3.0 T MRI scanner (MAGNETOM Skyra; Siemens Healthcare, Erlangen, Germany). To identify lesions and assess morphological traits, we first administered conventional MRI series such as spin-echo T1-weighted imaging (T1WI), T2-weighted imaging (T2WI), and fat-suppressed T2WI. We used multi-flip angle T1-weighted imaging technology to obtain T1 relaxation times (TRs) at three different flip angles (5°, 10°, and 15°) before contrast injection. Table S1 displays the MRI sequence parameters. T1WI three-dimensional volumetric interpolated breath-hold examination sequence was employed for DCE - MRI scans. The DCE images were taken on axial plane. The total acquisition time of VIBE sequence is 320 s, and the time resolution of each scan is 8 s; a total of 40 scans are obtained. In order to maintain a stable injection rate, we used an auto-injector for intravenous injection of gadoteridol (ProHance; Eisai, Tokyo, Japan) at a dose of 0.1mmol/kg at a rate of 2mL/s. Following that, at the same pace, we administered 20mL of physiological saline.

### MRI morphologic characteristics

MR images on the picture archiving and communication system (PACS) (m-view v5.4.10.71, INFINITT Healthcare) were independently evaluated by two experienced radiologists (with 5 and 8 years of diagnostic practice) who were blinded to clinical information and histopathological reports. Consensus was reached on the MRI morphological data. The following was recorded: (1) size(maximum diameter of tumor); (2) location(head and neck, trunk, upper limb, lower limb); (3) shape (non-multilobulated or multilobulated); (4) margin (well-defined or ill-defined); (5) lesion internal enhancement pattern (homogeneous or heterogeneous); (6) tumor necrosis; (7) peri-tumoral edema. The margin of a mass that was clearly separated from surrounding structures, regardless of neighboring peritumoral edema, was called a “well-defined” tumor. Heterogeneous, defined as the presence of areas of low, intermediate, and high signal intensity in ≥ 50% of the tumor volume. High signal on T2-weighted imaging without enhancement was considered evidence of tumor necrosis. Peritumoral edema is characterized as a fluid-like, high signal in the peritumoral region on T2WI that can be distinguished from the tumor entity. Labels 6 and 7 were classified as ‘yes’ or ‘no’.

### DCE-MR image analysis

We included time-signal intensity curve (TIC) types and quantitative evaluations in the DCE-MRI data analysis. Based on the previously described approach, the TIC types were characterized as having no evident upward trend or consistently increasing (type I), rapidly increasing then flattening (type II), or rapidly increasing and dropping (type III) [22]. The region of interest (ROI) of the DCE-MRI image was positioned in the solid portion of the lesion based on regular MRI findings and enhancing features of the lesion. According to the size of the solid portions of the lesion, the ROIs spanned areas of change ranging from 1 to 5 cm^2^. Necrotic, cystic, and hemorrhagic areas were avoided when drawing the ROIs. For each patient, two experienced radiologists manually chose ROIs in four typical slices, and the average value was determined as the parameter value. Image processing was conducted using commercially accessible software (MItalytics, FITPU Healthcare, Singapore). The software allows for the selection of a unique arterial input function for each patient case and employs a constrained nonlinear optimization approach to match the different models. In total, we obtained 25 independent and derived qDCE parameters. The TOFTS model was used to derive the following parameters: K^trans^ (min^− 1^), reverse reflux rate constant (K_ep_; min^− 1^), and extravascular extracellular volume (V_e_; mL/mL). Similarly, the EXTOFTS model was used to calculate K^trans^, K_ep_, V_e_, and volume fraction of plasma (V_p_; mL/mL). The ATH, CC, and DP model were used to obtain the parameters of V_e_, V_p_, F (mL/min/mL), PS (mL/min/mL), mean transit time (MTT; s), and extraction fraction (E; %). Supplementary Material A1 mathematically describes the parameter fitting with the equations of the tissue concentration-time curve *Ctiss(t)* used to fit pharmacokinetic models.

### Statistical analysis

All statistical analyses were conducted using R4.2.1 (www.r-project.org) and SPSS (version 25.0; SPSS, Chicago, III, USA).

MR morphologic characteristics and TIC types: The Shapiro-Wilk test was used to assess the normal distribution. The variables relating to benign and malignant lesions were assessed using the Mann-Whitney U test, χ2 test and univariate logistic regression analysis. Subsequently, we introduced the MRI morphological features and TIC with *P* < 0.05 into a multivariate logistic regression. The results of *P* < 0.05 were regarded as significant, and the findings were incorporated in the construction of traditional imaging model and subsequent studies.

Construction of qDCE models: We constructed the TOFTS, EXTOFTS, ATH, CC, and DP models using multivariate logistic regression based on the qDCE parameters derived from each TK model.

Development of top-parameter model: The Mann-Whitney U test, t test, and univariate logistic regression analysis were used to evaluate the qDCE parameters related to benign and malignant STTs. To filter the optimal parameters, we firstly attempted to incorporate all qDCE parameters of TK models into multifactor logistic regression. The variance inflation factor(VIF) was used to analyze multicollinearity. Some TK models have the same parameters or different TK model parameters interact with each other, multicollinearity is high, and some VIF values exceed 10. Therefore, the qDCE parameters with univariate logistic regression *P* < 0.05 were included in multiple logistic regression analysis with each TK model as the unit. The qDCE parameters with *P* < 0.05 were considered the top-parameters associated with the differentiation of benign and malignant STTs. There is no multicollinearity between top-parameters, and VIF < 10. Finally, we used the top-parameters to create a top-parameter model.

Construction and evaluation of the DP + Traditional imaging model and the comprehensive model: (1) The DP + Traditional imaging model was constructed using all parameters of the DP model and statistically significant traditional imaging features. (2) For the purpose of building the comprehensive model, we employed the top-parameters and statistically significant morphological features as input components. The area under the curve (AUC) was used to evaluate diagnostic performance. We also evaluated the accuracy, sensitivity, and specificity of each TK model and qDCE parameter.

## Results

The final diagnoses were based on histopathology, which showed 31 benign and 61 malignant lesions. Table 1 describes the detailed histopathological information regarding the two groups. Figure S1 shows pseudo-color images of an undifferentiated pleomorphic sarcoma. Figure S2 shows pseudo-color images of a schwannoma.

**Table 1:** Summary of classification of soft tissue tumors as per revised 2020 WHO criteria

Tumor classification	Benign tumors	Malignant tumors
Adipocytic	--	Myxoid liposarcoma (*n* = 6) Dedifferentiated liposarcoma (*n* = 2)
Fibroblastic/ myofibroblastic	Nodular fasciitis (*n* = 1) Proliferative myositis (*n* = 1) Fibroma of tendon sheath (*n* = 1) Elastofibroma (*n* = 2) Angiofibroma (*n* = 1)	Fibrosarcoma (*n* = 14) Myxofibrosarcoma (*n* = 9) Malignant solitary fibrous tumor (*n* = 2)
Peripheral nerve sheath	Schwannoma (*n* = 17) Neurofibroma (*n* = 2)	--
Vascular	Hemangioma (*n* = 5)	--
So-called fibrohistiocytic	Tenosynovial giant cell (*n* = 1)	--
Skeletal muscle	--	Rhabdomyosarcoma (*n* = 1)
Chondro-osseous	--	Extraskeletal osteosarcoma (*n* = 4)
Uncertain differentiation	--	Synovial sarcoma (*n* = 5) Epithelioid sarcoma (*n* = 1) Alveolar soft part sarcoma (*n* = 1) Undifferentiated pleomorphic sarcoma (*n* = 12)
Smooth muscle	--	Leiomyosarcoma (*n* = 4)

**Fig. 1:**
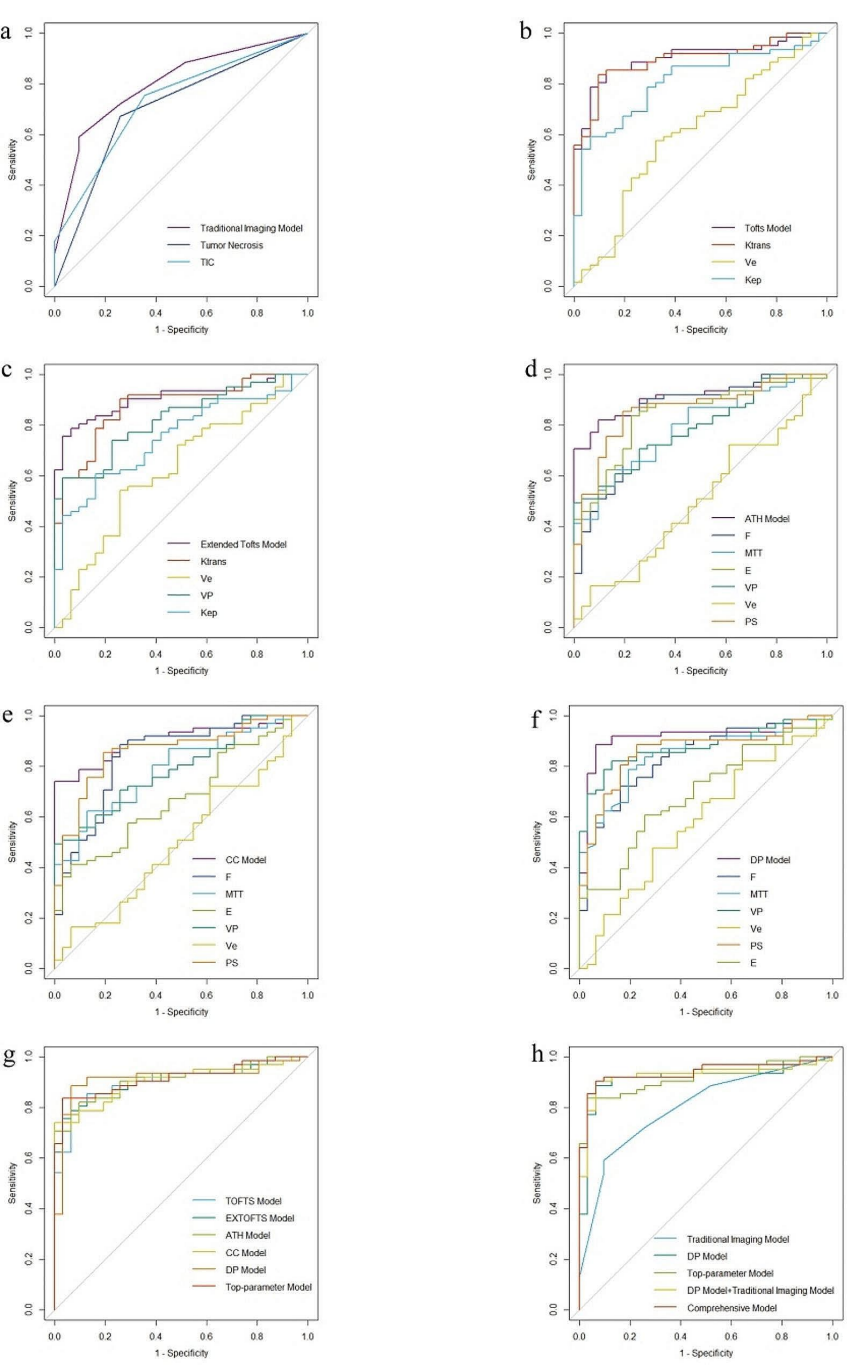
AUC of traditional imaging model (**a**), Tofts model (**b**), Extended Tofts model (**c**), ATH model (**d**), CC model (**e**), DP model (**f**), a comparison of five TK models and top-parameter model (**g**), a comparison of traditional imaging model, DP model, top-parameter model, DP model + traditional imaging model and comprehensive model (**h**)

Table 2 lists the relevant MRI morphological parameters and TIC. There were remarkable differences in shape, margin, tumor necrosis, peri-tumoral edema and TIC between the two groups. Univariate and multivariate analysis demonstrated that tumor necrosis (AUC 0.707), and TIC types (AUC 0.732) were independent predictors of benign and malignant STTs differentiation. Table S2 shows the results of univariate and multivariate analysis of morphological, TIC and qDCE parameters. Table S3 displays the diagnostic values of these parameters for separating malignant from benign STTs. Figure 1a shows the ROCs for MRI morphological parameters.

**Table 2:** MR Morphological features and TIC types

Variables	Benign(*n* = 31)	Malignant(*n* = 61)	*P* value
Size(mm) ^a^	57.0(32.4, 81.3) (range7.6-165.7)	70.3(43.2, 111.0) (range14.7-308.7)	0.09^#^
Location			
Head and neck^b^	6( 19.4%)	4( 6.6%)	0.19*
Trunk^b^	6( 19.4%)	8( 13.1%)	
Upper limb^b^	6( 19.4%)	13( 21.3%)	
Lower limb^b^	13( 41.9%)	36( 59.0%)	
Shape			0.011*
Non-multilobulated ^b^	26(83.9%)	35(57.4%)	
Multilobulated ^b^	5(16.1%)	26(42.6%)	
Margin			0.008*
Well-defined ^b^	25(80.6%)	32(52.5%)	
Ill-defined ^b^	6(19.4%)	29(47.5%)	
Enhancement pattern			0.70*
Homogeneous ^b^	5(16.1%)	8(13.1%)	
Heterogeneous ^b^	26(83.9%)	53(86.9%)	
Tumor necrosis			< 0.001*
Yes ^b^	8(25.8%)	41(67.2%)	
No ^b^	23(74.2%)	20(32.8%)	
Peri-tumoral edema			0.004*
Yes ^b^	18(58.1%)	52(85.2%)	
No ^b^	13(41.9%)	9(14.8%)	
TIC types			< 0.001*
Type I ^b^	20(64.5%)	15(24.6%)	
Type II ^b^	11(35.5%)	35(57.4%)	
Type III ^b^	-	11(18.0%)	

#Mann-Whitney U-test; * χ2 test. ^a^ Data are median (confidence interval); ^b^ Data are number of lesions, with percentage in parentheses. TIC time-signal intensity curve

Table 3 provides the statistical analysis of the qDCE parameters for benign and malignant STTs. There was no statistically significant difference in Ve values of all TK models between malignant and benign STTs (*P* > 0.05), while the differences in the other parameters were statistically significant (*P* < 0.05). Figure 1b-f shows the ROCs for qDCE parameters. Table S3 displays the optimal cutoff values and diagnostic performance for each parameter. TOFTS-K^trans^ achieved the greatest accuracy (0.859) and highest AUC (0.893) in predicting STTs.

**Table 3:** QDCE parameters derived with the TOFTS, EXTOFTS, ATH, CC and DP models

Parameters	Benign(*n* = 31)	Malignant(*n* = 61)	*P* value
TOFTS			
K^trans^ (min^− 1^) ^a^ K_ep_ (min^− 1^) ^a^ V_e_^b^	0.072(0.045,0.095) 0.258(0.193,0.365) 0.315 ± 0.146	0.168(0.126,0.244) 0.533(0.331,0.974) 0.365 ± 0.142	< 0.001* < 0.001* 0.117^#^
EXTOFTS			
K^trans^ (min^− 1^) ^a^ K_ep_ (min^− 1^) ^a^ V_e_^a^ V_p_^a^	0.067(0.040,0.090) 0.259(0.184,0.348) 0.248(0.202,0.396) 0.008(0.004,0.014)	0.142(0.100,0.214) 0.419(0.278,0.641) 0.345(0.234,0.459) 0.028(0.012,0.039)	< 0.001* < 0.001* 0.052* < 0.001*
ATH			
F ^a^ PS ^a^ V_e_^a^ V_P_^a^ MTT^a^ E ^a^	0.381(0.261,0.438) 0.061(0.041,0.076) 0.333(0.219,0.538) 0.007(0.004,0.016) 1.329(0.843,2.234) 15.095(12.267,18.466)	0.530(0.457,0.641) 0.153(0.102,0.222) 0.339(0.214,0.477) 0.032(0.008,0.061) 3.666(1.811,7.578) 25.361(20.442,30.462)	< 0.001* < 0.001* 0.98* < 0.001* < 0.001* < 0.001*
CC			
F ^a^ PS ^a^ V_e_^a^ V_P_^a^ MTT^a^ E ^a^	0.096(0.067,0.178) 0.080(0.059,0.162) 0.476(0.288,0.774) 0.008(0.005,0.023) 5.500(4.362,7.957) 58.154(34.785,80.590)	0.408(0.223,0.741) 0.149(0.101,0.272) 0.324(0.205,0.534) 0.065(0.028,0.096) 7.866(6.323,11.086) 37.230(20.710,69.214)	< 0.001* 0.001* 0.079* < 0.001* 0.001* 0.006*
DP			
F ^a^ PS ^a^ V_e_^a^ V_P_^a^ MTT^a^ E ^a^	0.147(0.096,0.236) 0.054(0.042,0.074) 0.402(0.256,0.591) 0.014(0.006,0.022) 3.907(3.000,7.591) 27.058(20.955,36.227)	0.344(0.238,0.476) 0.148(0.108,0.214) 0.318(0.216,0.492) 0.060(0.034,0.074) 10.000(8.270,12.096) 37.927(26.640,53.713)	< 0.001* < 0.001* 0.182* < 0.001* < 0.001* 0.002*

#t test; * Mann-Whitney U test. a Data are median (confidence interval); b Data are mean ± SD. K^trans^ transfer constant, K_ep_ reverse reflux rate constant, V_e_ extravascular extracellular volume, V_p_ volume fraction of plasma, F blood flow, PS permeability surface area product, MTT mean transit time, E extraction fraction. K^trans^ and K_ep_ are in units of min^− 1^, Ve and Vp are in units of mL/mL, F and PS are in units of mL/min/mL, MTT is in unit of seconds, E is in unit of %

Table 4 provides an illustration of the diagnostic performance of the nine models. The AUCs (0.899–0.915) of TOFTS, EXTOFTS, ATH, CC and DP models were all high, and the AUC(0.915) and accuracy (0.902) of the DP model was the highest. TOFTS-K^trans^ (AUC 0.893), EXTOFTS-K^trans^ (AUC 0.873), EXTOFTS-V_p_ (AUC 0.822), CC-V_p_ (AUC 0.870), and DP-V_p_ (AUC 0.875) were independent predictors of malignancy. The top-parameter model was constructed by TOFTS-K^trans^, EXTOFTS-K^trans^, EXTOFTS-V_p_, CC-V_p_ and DP-V_p_. The AUC value of the top-parameter model (0.914) is similar to DP model (0.915), but the AUC value of the comprehensive model, which is the optimal parameter model plus imaging features, is higher than that of the DP plus imaging features. The comprehensive model had the highest AUC (0.936, 95% CI, 0.884–0.988) among all the models. The ROCs of the five TK models and the top-parameter model are displayed in Fig. 1g.

**Table 4:** ROC analyses of the nine models established by logistic multiunivariate binary logistic analysis

Model	AUROC (95%CI)	Sensitivity (95%CI)	Specificity (95%CI)	Accuracy (95%CI)
Traditional imaging	0.802 (0.712–0.891)	0.590 (0.467–0.714)	0.903 (0.799-1.000)	0.696 (0.691-0.700)
TOFTS	0.899 (0.835–0.963)	0.852 (0.763–0.941)	0.871 (0.753–0.989)	0.859 (0.856–0.861)
EXTOFTS	0.904 (0.843–0.965)	0.787 (0.684–0.890)	0.935 (0.849-1.000)	0.837 (0.834–0.840)
ATH	0.909 (0.851–0.967)	0.820 (0.723–0.916)	0.903 (0.799-1.000)	0.848 (0.845–0.851)
CC	0.903 (0.841–0.964)	0.738 (0.627–0.848)	1.000 (1.000–1.000)	0.826 (0.823–0.829)
DP	0.915 (0.850–0.980)	0.885 (0.805–0.965)	0.935 (0.849-1.000)	0.902 (0.900-0.904)
Top-parameter	0.914 (0.856–0.971)	0.836 (0.743–0.929)	0.968 (0.906-1.000)	0.880 (0.878–0.883)
DP + Traditional imaging	0.925 (0.865–0.984)	0.902 (0.827–0.976)	0.935 (0.849-1.000)	0.913 (0.911–0.915)
Comprehensive	0.936 (0.884–0.988)	0.902 (0.827–0.976)	0.935 (0.849-1.000)	0.913 (0.911–0.915)

Traditional imaging model, morphological parameters and TIC types were chosen; TOFTS model, TOFTS transfer constant (K^trans^), TOFTS reverse reflux rate constant (K_ep_), TOFTS extravascular extracellular volume (V_e_) were chosen; EXTOFTS model, EXTOFTS-K^trans^, EXTOFTS-K_ep_, EXTOFTS-V_e_, EXTOFTS volume fraction of plasma (V_p_) were chosen; ATH, CC and DP model, blood flow (F), permeability surface area product (PS), V_e_, V_p_, mean transit time (MTT) and extraction fraction (E) were chosen; DP + Traditional imaging model, qDCE parameters (DP-F, DP-PS, DP-V_e_, DP-V_p_, DP-MTT, DP-E), morphological parameters, TIC types were chosen; Top-parameter model, the optimal quantitative dynamic contrast-enhanced MRI parameters (TOFTS-K^trans^, EXTOFTS-K^trans^, EXTOFTS-V_p_, CC-V_p_, DP-V_p_) were chosen; Comprehensive model, the optimal quantitative dynamic contrast-enhanced MRI parameters, morphological parameters and TIC types were combined together. AUROC area under the receiver operating characteristic curve; CI confidence interval

**Fig. 2:**
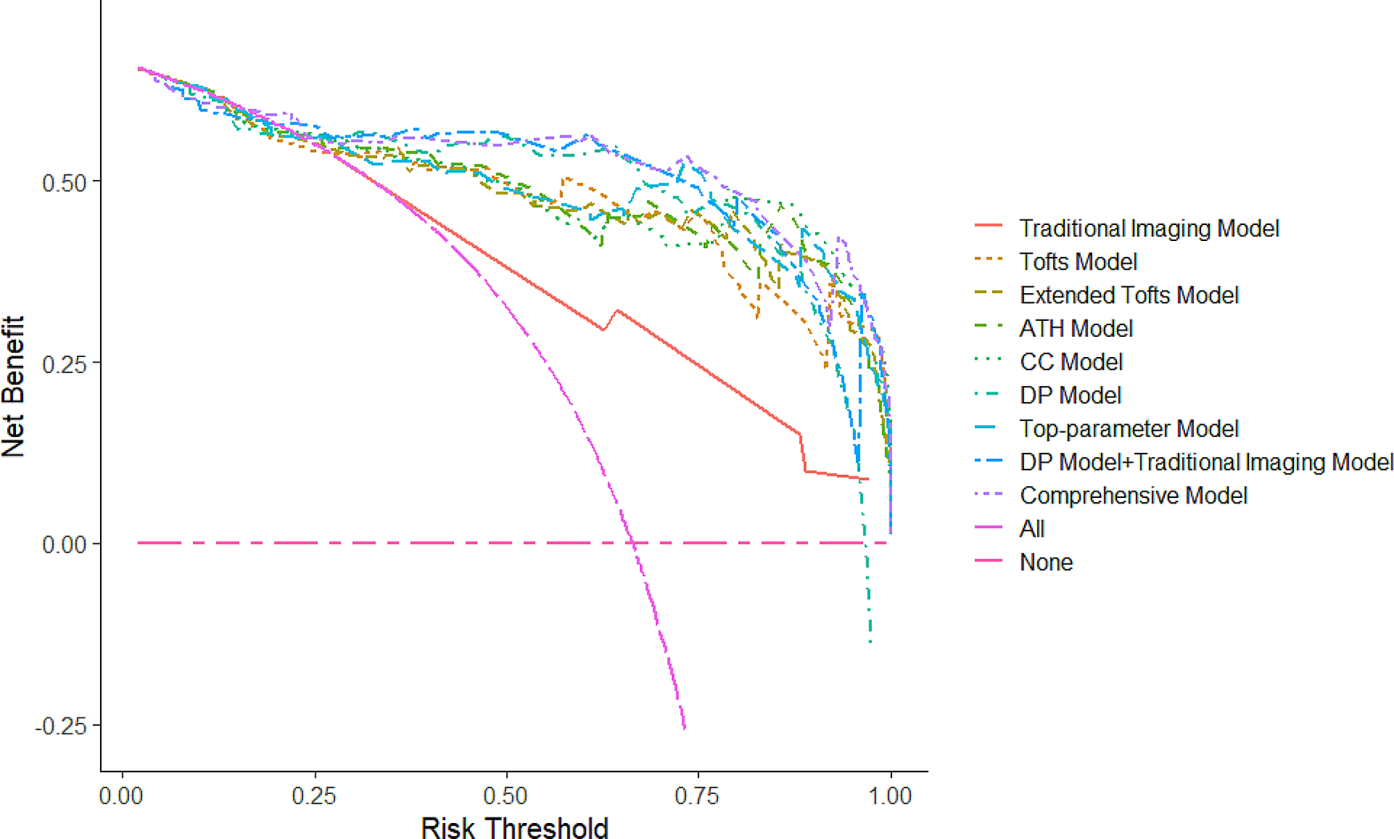
Decision curve analysis (DCA) of the nine models. The DCA indicated that the comprehensive model was more beneficial than the traditional imaging model and all individual quantitative dynamic contrast-enhanced MRI models when the threshold probability is between 0.1 and 1

Figure 2 illustrates the decision curve analysis (DCA) plot for the comprehensive model. It demonstrates that for a treatment threshold probability between 0.1 and 1, the comprehensive model outperformed the traditional imaging model including “treat none” versus “treat all” strategies.

## Discussion

This research assessed the efficacy of various TK models for categorizing of benign and malignant STTs. We demonstrate that they all accurately distinguish between such lesions (AUC 0.899–0.915). The multivariate binary logistic regression study revealed that TOFTS-K^trans^, EXTOFTS-K^trans^, EXTOFTS-V_p_, CC-V_p_, and DP-V_p_ are independent predictors of malignancy. We thus created a comprehensive model including these five qDCE parameters combined with tumor necrosis and TIC. This proposed comprehensive model is superior in differentiating between benign and malignant STTs. The comprehensive model’s AUC of 0.936 demonstrates that it outperforms the traditional imaging model (AUC 0.802) and the pharmacokinetic models (AUC 0.899–0.915). The DCA of the comprehensive model was likewise found to be more clinically beneficial than the traditional imaging and pharmacokinetic models.

DCE-MRI, a functional MRI technique, obtains T1-weighted magnetic resonance images dynamically after the injection of a considerable amount of contrast agent [23]. QDCE-MRI analysis fits different TK models to dynamically-acquired tissue concentration curves to enable the estimation of quantitative tissue parameters related to vascularity [10]. QDCE-MRI is promising in its ability to fundamentally describe the tumor’s vascularity and permeability. In our study, we individually evaluated the qDCE parameters obtained from the TOFTS, EXTOFTS, ATH, CC, and DP models. Among the parameters in all TK models, K^trans^ demonstrated the best diagnostic performance. The K^trans^ value represents the rate at which the contrast medium moves from the intravascular to the extravascular extracellular area. An elevated angiogenesis rate in malignant tumors causes a rise in blood flow and microvascular permeability, thus producing a greater K^trans^ value [14]. V_p_ is another suitable parameter to distinguish benign from malignant STTs, characterizing the degree of tissue microvascularity [16, 19]. Angiogenesis provides the necessary oxygen and nutrients for tumor growth and is closely associated to the malignant tumor growth [14, 24, 25]. Early studies revealed that the microvascular density of malignant STTs was significantly greater than that of benign STTs [26]. Our findings indicated that the V_p_ values of EXTOFTS, ATH, CC, and DP models in malignant lesions were substantially larger than those in benign lesions, consistent with previous findings. The parameter K^trans^ describes the combined processes of vascular permeability and tumor blood flow, but it is not clear which process is primarily responsible for K^trans^. In recent years, blood flow (F) and permeability-surface area product (PS) were used to describe intravascular perfusion and exchange between the intravascular and extravascular spaces respectively [10, 16, 20]. Unlike K^trans^ in the classical TK model, F and PS in the CC, ATH, DP models allow the permeability, PS, and the plasma blood flow, Fp to be estimated separately. Our results indicate that F and PS values — estimated by the CC, ATH, and DP models — are higher in malignant STTs. This may be because F and PS are related to the supply of nutrients necessary for cell growth, and these parameters are usually elevated because of the excessive proliferation of tumor cells [19].

Applying DCE-MRI for a combined measurement of perfusion and permeability necessitates using a suitable TK model to distinguish the contribution of both spaces [23]. Many compartmental and spatially distributed models have been proposed in the last two decades. The distinction between them is rooted in their assumption of lumped or distributed parameter compartments [10, 27]. The EXTOFTS model, a compartmental model, is the most common model for STTs [13–15, 26, 28]. In recent years, new TK models, such as ATH, CC, and DP, have been applied for a variety of diseases [16, 18, 20, 27]. These models consider both the spatial and temporal variations of an administered contrast agent, which is more realistic and likely to achieve greater accuracy and additional information than classical models .

Previous studies have demonstrated that ATH, CC, and DP models are effective in diagnosing cervical cancer tissue and providing additional information on glioma vessel permeability [16, 18, 19]. Our study analyzed all five TK models and found that the diagnostic performance of these models was similar. The DP model achieves the highest AUC and the best accuracy among all TK models. This may be because the DP model can describe blood flow and vascular permeability separately, avoiding the problem of confusing the two due to changes in the tissue microenvironment in the classical TK models. Compared with the TOFTS model, DP model introduced the parameters V_p_ and mean transit time(MTT) to characterize the degree of tissue microvessels. The description of more dimensions of tumor blood supply and complex calculations may make the results more accurate. In addition, we produced a comprehensive model combining the traditional imaging model and optimal parameter model to differentiate between benign and malignant STTs. The results show that the comprehensive model achieves the best differential diagnosis ability among all the models. Thus, our comprehensive model incorporating a vast array of multi-scale information more accurately reflects the blood circulation characteristics of tumors, allowing for a more reliable distinction between benign and malignant lesions. The promising outcome of this study encourages further research to formalize TK modelling and DCE-MRI as a possible imaging technique for preoperative risk assessment in patients with STTs.

There are several limitations to improve in future work. Firstly, this was a prospective study on a limited number of patients, and the findings should be confirmed for a larger sample. Secondly, given the low incidence of STTs and the diversity of their histopathological types, this study included STTs with different histological diagnoses. Thirdly, while the delineation of the ROI in this study avoided tumor necrosis and peritumoral edema, including only the solid part of the lesion, it could still be influenced by potential human error.

## Conclusion

ATH, CC, and DP have shown promise for STT DCE-MRI data and microcirculation pattern circulation analysis, and suitably supplement TOFTS and EXTOFTS-TOFTS. QDCE-MRI parameters effectively distinguish between malignant and benign STTs, particularly TOFTS-K^trans^, EXTOFTS-K^trans^, EXTOFTS-V_p_, CC-V_p_, and DP-V_p_. Combining qDCE-MRI data, morphological features, and kinetic curve types may result in superior diagnostic accuracy.

### Electronic supplementary material

Below is the link to the electronic supplementary material.

Supplementary Material 1

## Data Availability

The datasets used and/or analysed during the current study are available from the corresponding author on reasonable request.

## References

[1] Marzi S, Stefanetti L, Sperati F, Anelli V (2016). Relationship between diffusion parameters derived from intravoxel incoherent motion MRI and perfusion measured by dynamic contrast-enhanced MRI of soft tissue tumors. NMR Biomed.

[2] Zhao F, Ahlawat S, Farahani SJ, Weber KL, Montgomery EA, Carrino JA (2014). Can MR imaging be used to predict tumor grade in soft-tissue. Sarcoma? Radiol.

[3] Chhabra A, Soldatos T (2012). Soft-tissue lesions: when can we exclude sarcoma?. AJR Am J Roentgenol.

[4] Wang H, Zhang J, Bao S, Liu J, Hou F, Huang Y (2020). Preoperative MRI-Based Radiomic Machine-Learning Nomogram May accurately distinguish between Benign and malignant soft-tissue lesions: a two-Center Study. J Magn Reson Imaging.

[5] Crombe A, Alberti N, Stoeckle E, Brouste V, Buy X, Coindre JM (2016). Soft tissue masses with myxoid stroma: can conventional magnetic resonance imaging differentiate benign from malignant tumors?. Eur J Radiol.

[6] Arkun R, Argin M (2014). Pitfalls in MR imaging of musculoskeletal tumors. Semin Musculoskelet Radiol.

[7] Fields BKK, Demirjian NL, Hwang DH, Varghese BA, Cen SY, Lei X (2021). Whole-tumor 3D volumetric MRI-based radiomics approach for distinguishing between benign and malignant soft tissue tumors. Eur Radiol.

[8] Noebauer-Huhmann IM, Amann G, Krssak M, Panotopoulos J, Szomolanyi P, Weber M (2015). Use of diagnostic dynamic contrast-enhanced (DCE)-MRI for targeting of soft tissue tumour biopsies at 3T: preliminary results. Eur Radiol.

[9] Sourbron SP, Buckley DL (2011). On the scope and interpretation of the Tofts models for DCE-MRI. Magn Reson Med.

[10] Khalifa F, Soliman A, El-Baz A, Abou El-Ghar M, El-Diasty T, Gimel’farb G (2014). Models and methods for analyzing DCE-MRI: a review. Med Phys.

[11] Koh TS, Bisdas S, Koh DM, Thng CH (2011). Fundamentals of tracer kinetics for dynamic contrast-enhanced MRI. J Magn Reson Imaging.

[12] Tofts PS, Berkowitz B, Schnall MD (1995). Quantitative analysis of dynamic Gd-DTPA enhancement in breast tumors using a permeability model. Magn Reson Med.

[13] Zhang Y, Yue B, Zhao X, Chen H, Sun L, Zhang X (2020). Benign or malignant characterization of soft-tissue tumors by using semiquantitative and quantitative parameters of dynamic contrast-enhanced magnetic resonance imaging. Can Assoc Radiol J.

[14] Choi YJ, Lee IS, Song YS, Kim JI, Choi KU, Song JW (2019). Diagnostic performance of diffusion-weighted (DWI) and dynamic contrast-enhanced (DCE) MRI for the differentiation of benign from malignant soft-tissue tumors. J Magn Reson Imaging.

[15] Lee SK, Jee WH, Jung CK, Chung YG (2020). Multiparametric quantitative analysis of tumor perfusion and diffusion with 3T MRI: differentiation between benign and malignant soft tissue tumors. Br J Radiol.

[16] Li Z, Zhao W, He B, Koh TS, Li Y, Zeng Y (2020). Application of distributed parameter model to Assessment of Glioma IDH Mutation Status by Dynamic contrast-enhanced magnetic resonance imaging. Contrast Media Mol Imaging.

[17] Sourbron SP, Buckley DL (2012). Tracer kinetic modelling in MRI: estimating perfusion and capillary permeability. Phys Med Biol.

[18] Shao J, Zhang Z, Liu H, Song Y, Yan Z, Wang X (2020). DCE-MRI pharmacokinetic parameter maps for cervical carcinoma prediction. Comput Biol Med.

[19] Wang X, Lin W, Mao Y, Peng W, Song J, Lu Y (2019). A comparative study of two-Compartment Exchange models for Dynamic contrast-enhanced MRI in characterizing uterine cervical carcinoma. Contrast Media Mol Imaging.

[20] Ye Z, Ning G, Li X, Koh TS, Chen H, Bai W (2022). Endometrial carcinoma: use of tracer kinetic modeling of dynamic contrast-enhanced MRI for preoperative risk assessment. Cancer Imaging.

[21] Moch HJWCoT. Soft Tissue and Bone Tumours WHO Classification of Tumours/Volume 3 2020;3.

[22] Li X, Xie Y, Hu Y, Lu R, Li Q, Xiong B (2022). Soft tissue sarcoma: correlation of dynamic contrast-enhanced magnetic resonance imaging features with HIF-1α expression and patient outcomes. Quant Imaging Med Surg.

[23] Sourbron SP, Buckley DL (2013). Classic models for dynamic contrast-enhanced MRI. NMR Biomed.

[24] Partridge SC, Demartini WB, Kurland BF, Eby PR, White SW, Lehman CD (2010). Differential diagnosis of mammographically and clinically occult breast lesions on diffusion-weighted MRI. J Magn Reson Imaging.

[25] Holash J, Maisonpierre PC, Compton D, Boland P, Alexander CR, Zagzag D (1999). Vessel cooption, regression, and growth in tumors mediated by angiopoietins and VEGF. Science.

[26] Zhang Y, Zhao H, Liu Y, Zeng M, Zhang J, Hao D (2022). Diagnostic performance of dynamic contrast-enhanced MRI and (18)F-FDG PET/CT for Evaluation of Soft Tissue Tumors and correlation with Pathology parameters. Acad Radiol.

[27] Lu Y, Peng W, Song J, Chen T, Wang X, Hou Z (2019). On the potential use of dynamic contrast-enhanced (DCE) MRI parameters as radiomic features of cervical cancer. Med Phys.

[28] Lee JH, Yoon YC, Seo SW, Choi YL, Kim HS (2020). Soft tissue sarcoma: DWI and DCE-MRI parameters correlate with Ki-67 labeling index. Eur Radiol.

