# Supplementary material for: Applying dynamic contrast-enhanced MRI tracer kinetic models to differentiate benign and malignant soft tissue tumors
Source: Cancer Imaging. 2024 May 21;24:64. doi: 10.1186/s40644-024-00710-x (PMC11107050; doi:10.1186/s40644-024-00710-x)
Supplement: Supplementary file 1 — Supplementary Material 1 [file 40644_2024_710_MOESM1_ESM.docx]

**Supplementary material**

**Supplementary A1: The equations of the tissue concentration-time curve *Ctiss(t)***

(1)TOFTS

$\frac{dC_{t}}{dt}=K^{trans}C_{p}\left( t \right)-K_{ep}C_{t}(t)=K^{trans}\left( C_{p}(t)-\frac{C_{t(t)}}{V_{e}} \right)$[1]

(2)EXTOFTS

$$C_{t}\left( t \right)=V_{P}C_{P}\left( t \right)+C_{P}\left( t \right) \bigotimes H_{TK}\left( t \right)$$

$=V_{P}C_{P}\left( t \right)+K^{trans}\int_{0}^{t} C_{p}(t^{'})e^{-\left( \left( K^{trans}/V_{e} \right)\left( t-t^{'} \right) \right)}dt^{'}$[2]

(3)ATH

$$A_{i}\frac{\partial C_{t}\left( x,t \right)}{\partial t}=-F\frac{\partial C_{t}\left( x,t \right)}{\partial x}-\frac{PS}{L}\left[ C_{t}(x,t)-\frac{C_{e}(t)}{\lambda} \right]$$

$A_{e}L\frac{dC_{e}(t)}{dt}=\frac{PS}{L}\int_{0}^{L} \left[ C_{t}(x,t)-\frac{C_{e}(t)}{\lambda} \right]dx$[3, 4]

(4)CC

$$v_{p}\frac{dC_{p}\left( t \right)}{dt}=-PS\left[ C_{p}\left( t \right)-C_{e}\left( t \right) \right]-F_{p}C_{p}\left( t \right)+C_{0}\delta\left( t \right)$$

$v_{e}\frac{dC_{e}\left( t \right)}{dt}=PS\left[ C_{p}\left( t \right)-C_{e}\left( t \right) \right]$[5]

(5)DP

$$v_{p}\frac{\partial C_{p}\left( x,t \right)}{\partial t}= -LF_{p}\frac{\partial C_{p}\left( x,t \right)}{\partial x}-PS\left[ C_{p}\left( x,t \right){-C}_{e}\left( x,t \right) \right]$$

$$v_{e}\frac{\partial C_{e}\left( x,t \right)}{\partial t}=PS\left[ C_{p}\left( x,t \right){-C}_{e}\left( x,t \right) \right]$$

and

$$H_{DP}\left( t \right)=u\left( t \right)-u\left( t-T_{c} \right)exp\left( \frac{PS}{F_{p}} \right)$$

$\times\left\{ 1+PS\int_{0}^{t-T_{c}} \left( \frac{1}{t^{'}F_{p}v_{e}} \right)^{0.5}e^{-\left( \left( PS/v_{e} \right)t' \right)}\times I_{1}\left( 2PS\left( {\frac{t^{'}}{F_{p}v_{e}}}^{0.5} \right) \right)dt^{'} \right\}$[6-8]

**TableS1.Parameters of DCE-MRI sequences**

|  | T1 mapping | DCE-MRI | T1WI | T2WI | FS-T2WI |
| --- | --- | --- | --- | --- | --- |
| TR(ms) | 5.17 | 5.17 | 650 | 2220 | 2220 |
| TE(ms) | 1.78 | 1.78 | 13 | 80 | 80 |
| Slice thickness(mm) | 4 | 4 | 3 | 3 | 3 |
| Flip angle | 5°,10°,15° | 15° | 120° | 120° | 120° |
| Matrix | 256×256 | 256×256 | 256×256 | 256×256 | 256×256 |
| Number of excitation | 1 | 1 | 2 | 2 | 2 |
| BW | 260 | 260 | 240 | 260 | 260 |
| ETL | - | - | 3 | 18 | 18 |
| PAT mode | GRAPPA, 2 | GRAPPA, 2 | GRAPPA, 2 | NO | NO |

TR, repetition time; TE, echo time; BW, bandwidth; ETL, echo train length; PAT, Parallel acquisition technique; DCE-MRI, Dynamic contrast-enhanced MRI; T1WI, T1-weighted imaging; T2WI, T2-weighted imaging; FS, fat suppression; GRAPPA, generalized autocalibrating partially parallel acquisition.

**TableS2.Results of univariate & multivariate logistic regression analysis for benign and malignant soft tissue tumors.**

| Variables | | Univariate | | *P* value | Multivariate | | *P* value |
| --- | --- | --- | --- | --- | --- | --- | --- |
|  |  | OR | 95%CI |  | OR | 95%CI |  |
| Size | | 1.01 | 1.00-1.02 | .07 |  | | |
| Location | | 2.00 | 0.38-10.41 | .21 |  | | |
| Shape | | 3.86 | 1.31-11.41 | .01 | 1.23 | 0.97-1.44 | .11 |
| Margin | | 3.78 | 1.36-10.50 | .01 | 0.98  0.25-4.74  0.9027 | 0.79-1.21 | .85 |
| Enhancement pattern | | 1.27 | 0.38-4.28 | .69 |  | | |
| Tumor  necrosis | | 5.89 | 2.24-15.48 | < .001 | 1.24 | 1.03-1.49 | .02* |
| Peri-tumoral  edema | | 4.17 | 1.53-11.40 | .005 | 1.17 | 0.94-1.44 | .16 |
| TIC | | 4.24 | 1.64-11.00 | .003 | 1.25 | 1.09-1.43 | .002* |
| TOFTS-K^trans^ | | 4.39e^13^ | 3.51*10^7^-5.49e^19^ | < .001 | 3.82e^15^ | 1.27e^7^-1.15e^24^ | < .001* |
| TOFTS-k_ep_ | | 237.00 | 10.72-5238.41 | .001 | 2.4e^-1^ | 4.66e^-3^-1.24e | .48 |
| TOFTS-V_e_ | | 13.48 | 0.51-356.28 | .12 |  | | |
| EX- K^trans^ | | 1.23e^15^ | 9.61*10^7^-1.57e^22^ | < .001 | 6.21e^12^ | 2.03e^4^-1.89e^21^ | .003* |
| EX- K_ep_ | | 193.68 | 7.00-5363.75 | .002 | 3.49e^-1^ | 6.51e^-3^-1.88e | .60 |
| EX- V_e_ | | 7.85 | 0.57-108.35 | .12 |  | | |
| EX- V_p_ | | 4.26e^48^ | 1.46e^25^-1.24e^72^ | < .001 | 1.15e^27^ | 5.58e^4-^2.35e^49^ | .02* |
| ATH-F | | 1.09*10^5^ | 403.59-2.94*10^7^ | < .001 | 2.07 | 0-7.44e3 | .86 |
| ATH-PS | | 01.01e^12^ | 2.61*10^6^-3.92e^17^ | < .001 | 1.61e^7^ | 0-2.1e^18^ | .20 |
| ATH-V_P_ | | 1.47e^28^ | 1.07e^13^  -2e^43^ | < .001 | 9.02e^23^ | 0.35-2.34e^48^ | .06 |
| ATH-V_e_ | | 1.41 | 0.18-10.88 | 1.41 |  | | |
| ATH-MTT | | 1.76 | 1.28-2.42 | .001 | 1.00 | 0.66-1.50 | .97 |
| ATH-E | | 1.23 | 1.12-1.35 | < .001 | 1.06 | 0.94-1.19 | .34 |
| CC-F | | 7.36*10^3^ | 84.15-6.44*10^5^ | < .001 | 4.11e^-2^ | 5.57e^-7^-3.02e^3^ | .58 |
| CC-PS | | 359.27 | 2.49-51797.60 | .02 | 4.69 | 3.06e^-3^-7.19e^3^ | .68 |
| CC-V_P_ | | 1.28e^28^ | 5.02e^14^-3.25e^41^ | < .001 | 5.50e^58^ | 1.37e^6^-2.20e^111^ | .03* |
| CC-V_e_ | | 0.23 | 0.05-1.16 | .08 |  | | |
| CC-MTT | | 1.30 | 1.10-1.54 | .003 | 7.70e^-1^ | 5.13e^-1^-1.15 | .21 |
| CC-E | | 0.98 | 0.96-0.99 | .01 | 1.03 | 9.89e^-1^-1.08 | .14 |
| DP-F | | 4.56*10^4^ | 313.36-6.64*10^6^ | < .001 | 2.77e^-4^ | 4.17e^-11^-1.84e^3^ | .31 |
| DP-PS | | 6.85e^11^ | 3.17*10^6^-1.48e^17^ | < .001 | 1.83e^2^ | 1.59e^-10^-2.10^14^ | .71 |
| DP-V_p_ | | 2.69e^31^ | 4.93e^17^-1.46e^45^ | < .001 | 3.84e^65^ | 1.95e^12^-7.58e^118^ | .02* |
| DP-V_e_ | | 0.30 | 0.05-2.00 | .21 |  | | |
| DP-MTT | | 1.50 | 1.27-1.77 | < .001 | 7.29e^-1^ | 4.64e^-1^-1.15 | .17 |
| DP-E | | 1.05 | 1.02-1.09 | .005 | 1.05 | 9.62e^-1^-1.15 | .27 |

TIC time-signal intensity curve; K^trans^ transfer constant; K_ep_ reverse reflux rate constant; V_e_ extravascular extracellular volume; V_p_ volume fraction of plasma; F blood flow; PS permeability surface area product; MTT mean transit time; E extraction fraction. K^trans^ and K_ep_ are in units of min^−1^, Ve and Vp are in units of mL/mL, F and PS are in units of mL/min/mL, MTT is in unit of seconds, E is in unit of %.

**TableS3.Diagnostic performance of morphological parameters, TIC type and qDCE parameters discrimination between malignant and benign lesions(n=92).**

| Parameters | Cutoff value | AUROC  (95％CI) | Sensitivity  (95% CI) | Specificity  (95% CI) | Accuracy  (95% CI) |
| --- | --- | --- | --- | --- | --- |
| Tumor necrosis | 0.500 | 0.707  (0.609-0.805) | 0.672  (0.554-0.790) | 0.742  (0.588-0.896) | 0.696  (0.691-0.700) |
| TIC | 1.500 | 0.732  (0.638-0.825) | 0.754  (0.646-0,862) | 0.645  (0.477-0.814) | 0.717  (0.713-0.722) |
| Tofts-K^trans^ | 0.116 | 0.893  (0.828-0.959) | 0.836  (0.743-0.929) | 0.903  (0.799-1.000) | 0.859  (0.856-0.861) |
| Tofts-K_ep_ | 0.457 | 0.808  (0.719-0.897) | 0.590  (0.467-0.714) | 0.935  (0.849-1.000) | 0.707  (0.702-0.711) |
| Tofts-V_e_ | 0.345 | 0.602  (0.476-0.729) | 0.574  (0.450-0.698) | 0.677  (0.513-0.842) | 0.609  (0.604-0.614) |
| Ex- K^trans^ | 0.080 | 0.873  (0.800-0.946) | 0.902  (0.827-0.976) | 0.742  (0.588-0.896) | 0.848  (0.845-0.851) |
| Ex- K_ep_ | 0.379 | 0.749  (0.650-0.849) | 0.607  (0.484-0.729) | 0.839  (0.709-0.968) | 0.685  (0.680-0.689) |
| Ex- V_e_ | 0.340 | 0.625  (0.501-0.748) | 0.541  (0.416-0.666) | 0.742  (0.588-0.896) | 0.609  (0.604-0.614) |
| Ex- V_p_ | 0.023 | 0.822  (0.739-0.905) | 0.590  (0.467-0.714) | 0.968  (0.906-1.000) | 0.717  (0.713-0.722) |
| ATH-F | 0.418 | 0.840  (0.752-0.929) | 0.885  (0.805-0.965) | 0.742  (0.588-0.896) | 0.837  (0.834-0.840) |
| ATH-PS | 0.082 | 0.860  (0,782-0.938) | 0.852  (0.763-0.941) | 0.806  (0.667-0.946) | 0.837  (0.834-0.840) |
| ATH-V_e_ | 0.252 | 0.502  (0.376-0.628) | 0.721  (0.609-0.834) | 0.387  (0.216-0.559) | 0.609  (0.604-0.614) |
| ATH-V_p_ | 0.034 | 0.788  (0.698-0.879) | 0.492  (0.366-0.617) | 1.000  (1.000-1.000) | 0.663  (0.658-0.668) |
| ATH-MTT | 2.927 | 0.790  (0.698-0.881) | 0.623  (0.501-0.745) | 0.871  (0.753-0.989) | 0.707  (0.702-0.711) |
| ATH-E | 18.649 | 0.839  (0.756-0.922) | 0.836  (0.743-0.929) | 0.774  (0.627-0.921) | 0.815  (0.812-0.818) |
| CC-F | 0.210 | 0.863  (0.788-0.938) | 0.820  (0.723-0.916) | 0.839  (0.709-0.968) | 0.826  (0.823-0.829) |
| CC-PS | 0.096 | 0.712  (0.597-0.828) | 0.787  (0.684-0.890) | 0.677  (0.513-0.842) | 0.750  (0.746-0.754) |
| CC-V_e_ | 0.953 | 0.388  (0.265-0.510) | 0.033  (-0.012-0.077) | 1.000  (1.000-1.000) | 0.359  (0.354-0.364) |
| CC-V_p_ | 0.040 | 0.870  (0.799-0.941) | 0.672  (0.554-0.790) | 0.968  (0.906-1.000) | 0.772  (0.768-0.775) |
| CC-MTT | 5.531 | 0.719  (0.608-0.830) | 0.852  (0.763-0.941) | 0.516  (0.340-0.692) | 0.739  (0.735-0.743) |
| CC-E | - | 0.324  (0.214-0.434) | 1.000  (1.000-1.000) | - | 0.337  (0.332-0.342) |
| DP-F | 0.265 | 0.838  (0.754-0.921) | 0.721  (0.609-0.834) | 0.839  (0.709-0.968) | 0.761  (0.757-0.765) |
| DP-PS | 0.075 | 0.858  (0.778-0.938) | 0.885  (0.805-0.965) | 0.774  (0.627-0.921) | 0.848  (0.845-0.851) |
| DP-V_e_ | 0.108 | 0.415  (0.289-0.540) | 0.984  (0.952-1.000) | 0.065  (-0.022-0.151) | 0.674  (0.669-0.679) |
| DP-V_p_ | 0.028 | 0.875  (0.804-0.945) | 0.820  (0.723-0.916) | 0.871  (0.753-0.989) | 0.837  (0.834-0.840) |
| DP-MTT | 7.878 | 0.846  (0.767-0.926) | 0.787  (0.684-0.890) | 0.806  (0.667-0.946) | 0.793  (0.790-0.797) |
| DP-E | 35.266 | 0.694  (0.585-0.804) | 0.607  (0.484-0.729) | 0.742  (0.588-0.896) | 0.652  (0.647-0.657) |

TIC time-signal intensity curve; K^trans^ transfer constant; K_ep_ reverse reflux rate constant; V_e_ extravascular extracellular volume; V_p_ volume fraction of plasma; F blood flow; PS permeability surface area product; MTT mean transit time; E extraction fraction. K^trans^ and K_ep_ are in units of min^−1^, Ve and Vp are in units of mL/mL, F and PS are in units of mL/min/mL, MTT is in unit of seconds, E is in unit of %. AUROC area under the receiver operating characteristic curve;

CI confidence interval. TIC type: Type I, Type II, Type III.

**
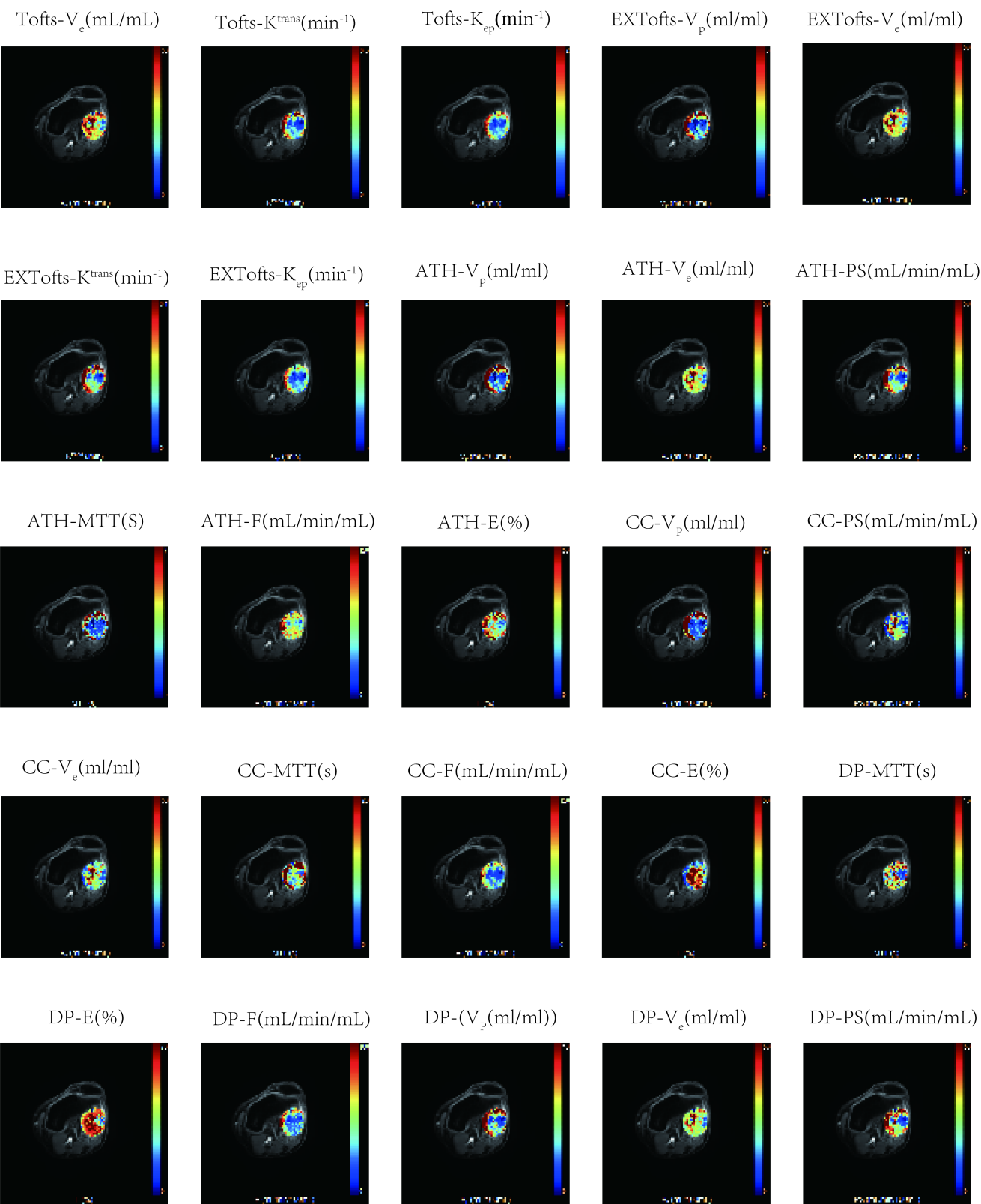
**

**Figure S1:** A 52-year male with malignant soft-tissue tumors (undifferentiated pleomorphic sarcoma). Parameter maps generated using the five models (TOFTS, EXTOFTS, ATH, CC, and DP) for tumor tissue ROIs.

**
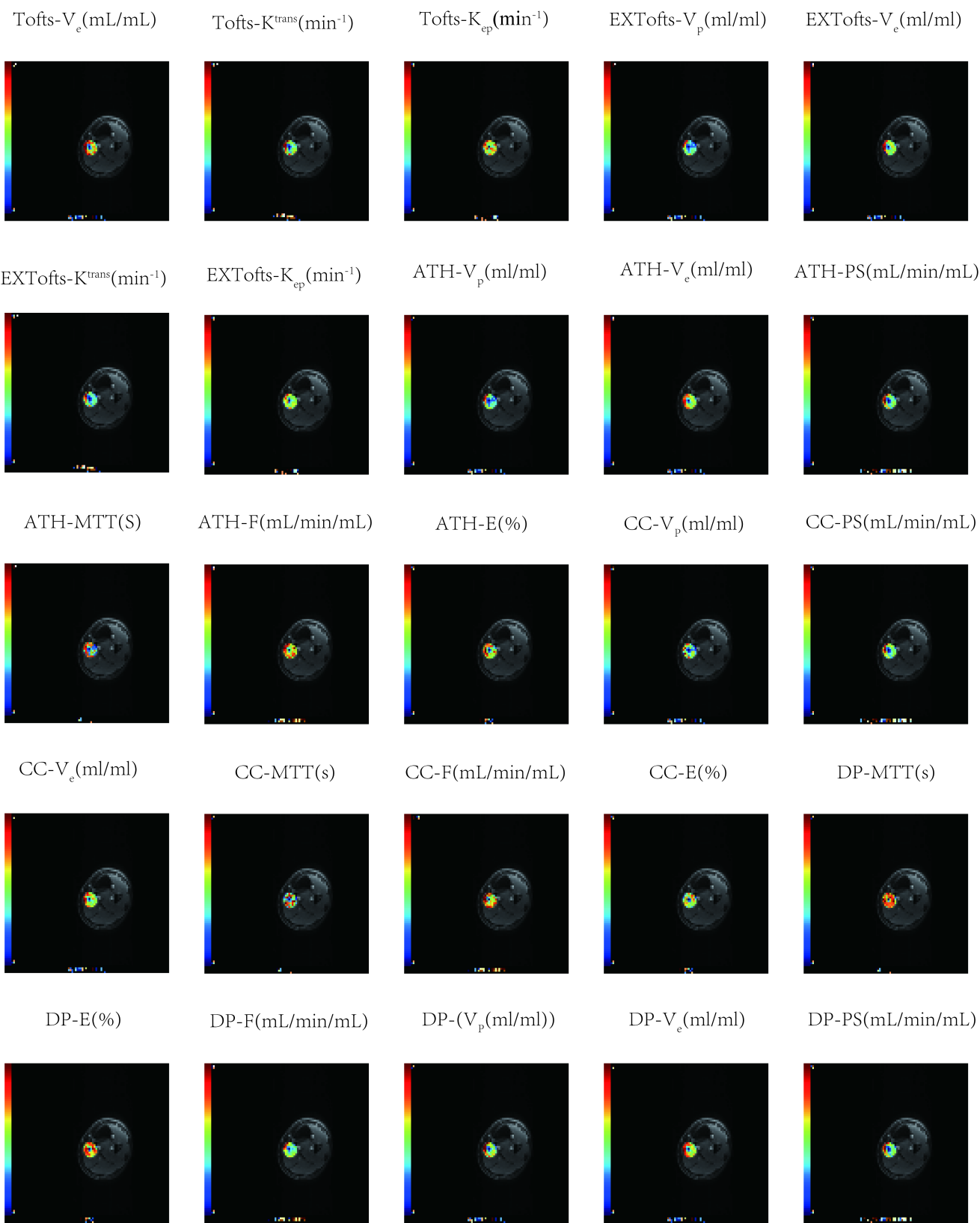
**

**Figure S2:** A 30-year male with benign soft-tissue tumors (schwannoma). Parameter maps generated using the five models (TOFTS, EXTOFTS, ATH, CC, and DP) for tumor tissue ROIs.

**Reference**

1. Liu H, Jiang Y, Dai Q, Zhu Q, Wang L, Zhang J, et al. Differentiation of benign and malignant sub-1-cm breast lesions using contrast-enhanced sonography. *J Ultrasound Med* 2015;34:117-123

2. Hylton N. Dynamic contrast-enhanced magnetic resonance imaging as an imaging biomarker. *J Clin Oncol* 2006;24:3293-3298

3. Lawrence KSS, Lee T-Y. An Adiabatic Approximation to the Tissue Homogeneity Model for Water Exchange in the Brain: I. Theoretical Derivation 1998;18:1365-1377

4. Lawrence KSS, Lee T-Y. An Adiabatic Approximation to the Tissue Homogeneity Model for Water Exchange in the Brain: II. Experimental Validation 1998;18:1378-1385

5. Koh TS, Bisdas S, Koh DM, Thng CH. Fundamentals of Tracer Kinetics for Dynamic Contrast-Enhanced MRI. *Journal of Magnetic Resonance Imaging* 2011;34:1262-1276

6. Positano V, Santarelli MF, Landini L. Automatic characterization of myocardial perfusion in contrast enhanced MRI. *Eurasip Journal on Applied Signal Processing* 2003;2003:413-421

7. Olafsdottir H, Stegmann MB, Larsson HBW. *Automatic assessment of cardiac perfusion MRI*. In: Barillot C, Haynor DR, Hellier P, eds. *Medical Image Computing and Computer-Assisted Intervention - Miccai 2004, Pt 2, Proceedings*, 2004:1060-1061

8. Xue H, Zuehlsdorff S, Kellman P, Arai A, Nielles-Vallespin S, Chefdhotel C, et al. Unsupervised Inline Analysis of Cardiac Perfusion MRI. In:*12th International Conference on Medical Image Computing and Computer-Assisted Intervention (MICCAI2009)*. Imperial Coll, London, ENGLAND, 2009; 741-+
